# The impact of environmental factors on the transport and survival of pathogens in agricultural soils from karst areas of Yunnan province, China: Laboratory column simulated leaching experiments

**DOI:** 10.3389/fmicb.2023.1143900

**Published:** 2023-03-16

**Authors:** Zhuo Ning, Shuaiwei Wang, Caijuan Guo, Min Zhang

**Affiliations:** ^1^Institute of Hydrogeology and Environmental Geology, Chinese Academy of Geological Sciences, Shijiazhuang, China; ^2^Key Laboratory of Groundwater Remediation of Hebei Province and China Geological Survey, Zhengding, China

**Keywords:** pathogenic microorganisms, transport and survival of pathogens, leaching experiments, agricultural soils, karst area

## Abstract

**Introduction:**

Groundwater is considered the best candidate for drinking water supply in the karst area. The groundwater water resources, however, are vulnerable to pathogenic microorganism contamination because of the typically thin soil layers overlying aquifers and the high permeability of the aquifer host rock, resulting in short residence times and low natural attenuation capacities. Until now, little attention has been paid to the critical environmental factors affecting the pathogenic microorganism contamination in soil-groundwater systems in the karst area.

**Methods:**

In the study, orthogonality column experiments with controlling ambient temperatures, pH values of inlet water, and soil porosities were carried out to investigate the transport and lifespan of pathogenic microorganisms in the leachate of agricultural soils in the karst area of Yunnan province, China. The pathogenic indicators, i.e., total bacteria count (TBC) and total coliforms count (TCC), and hydrochemical parameters, i.e., pH and permanganate index (COD_Mn_) in the leaching water, were systematically monitored.

**Results and Discussion:**

The results showed that bacteria including coliforms can survive for prolonged periods of time in karst soils. The soils overlying the karst rocks were unable to impede the bacteria from seeping into the groundwater. The soils, in turn, likely served as both reservoirs and incubators for pathogenic bacteria. The ambient temperature was the most predominant influential factor affecting both TBC and TCC. The bacteria concentrations were proportional to the temperature in the leachate. Therefore, more attention should be paid to temperature variations in protecting the water supply, particularly in the high-temperature period, such as during the summer months.

## 1. Introduction

Groundwater is the best candidate for rural water supply in the karst area of Southwest China (Xia, 2016). Unfortunately, these important water resources are vulnerable to anthropogenic contamination. Because of the typically thin vadose zone soil layers and the high permeability of the host rock, the short residence time and low natural attenuation capacities may lead to the formation of infertile soils in the thin layers and the enrichment of contaminants in groundwater. Soil, water, nutrients, and contamination are easily leached *via* underground channels or conduits created by the widening of fractures in soluble rocks such as limestone or dolomite (Heinz et al., 2009). In agricultural soils, manure is frequently used to improve soil fertility. The pathogens embedded in the manure could concurrently be transferred into soils and then leach into the groundwater (Franz et al., 2008; Sharma and Reynnells, 2018; Pang et al., 2020; Chique et al., 2021). Studies have shown that pathogenic microorganisms are significant sources of anthropogenic contamination in rural villages, potentially posing a risk to water supply security, particularly in karst regions. This is due to the fact that pathogen infections are typically linked to the consumption of groundwater contaminated with pathogens (Heinz et al., 2009; Luffman and Tran, 2014; Li et al., 2020; Brad et al., 2022).

Transport of pathogens through soils is governed by several basic physical processes such as advection, dispersion, adhesion/detachment, as well as survival process growth/decay (Bitton and Harvey, 1992; Ginn et al., 2002; Sen, 2011). Most studies focused on the physical processes in the aquifer or the vadose zone relying on the column experiments (McCaulou et al., 1994; Tan et al., 1994; McCaulou et al., 1995; Schäfer et al., 1998; Ginn et al., 2006; Liu et al., 2011; Aaron, 2018; Eisfeld et al., 2022). Several studies also paid attention to the survival of pathogens in various soils or groundwater (Edmonds, 1976; Bitton et al., 1983; John and Rose, 2005; Grisey et al., 2010; Chandrasekar et al., 2021). These studies revealed that the transport and survival of pathogens are mainly affected by four factors: climate (e.g., temperature, rainfall), medium materials and conditions (e.g., texture, pH, water holding capacity, cation exchange capacity), properties of fluids (e.g., chemistry, saturation), and type of pathogen (e.g., Bacteria, fungi, protozoa, virus) (Bitton and Harvey, 1992; Franz et al., 2008; Dwivedi et al., 2016; Song et al., 2018; Pang et al., 2020). Some studies also indicate that the survival and growth of some bacteria are greatly affected by the protozoa in the soil or groundwater (King et al., 1988; England et al., 1993; Matz and Kjelleberg, 2005; Lambrecht et al., 2015).

In the karst area, previous research mainly focused on the origin of the pathogens and pointed out that agricultural activity would introduce pathogens to the karst aquifer (Pronk, 2008; Heinz et al., 2009). The survival and transport of several pathogens in various karstic aquifers have been extensively studied and found that once the pathogenic microorganisms penetrated the soil and entered into the karst aquifer, the microorganisms may migrate fast through karst channels, especially in Southwest China (Personné et al., 1998; Pronk et al., 2006; Pronk, 2008; Ward et al., 2016; Bandy et al., 2018; Buckerfield et al., 2019; Bandy et al., 2020; Oliver et al., 2020). Infiltration in the vadose zone is crucial in preventing and regulating the contamination of groundwater by pathogens. In South-Western Karst Region, most of the soils stem from limestone weathering, thus their available element contents are low, and clay and organic matter contents are high (Cao et al., 2003; Li et al., 2006; Chen and Bi, 2011). In agricultural soils, the movement and persistence of pathogens and related elements may differ from those found in typical soils. However, there have been limited studies on this subject. In our previous research, we discovered that pH, temperature, and porosity are the significant factors that influence the presence of pathogens, specifically the total bacteria count (TBC) and the total coliform count (TCC) (Wang, 2019). It is important to study the impact of these factors on the movement and survival of pathogens.

In this study, laboratory column simulated leaching experiments, with agricultural soils from karst areas of Yunnan province, China, were carried out. By altering the soil porosity, the environmental temperature, as well as the inlet water pH values, the variations of pathogenic bacteria (represented by TBC and TCC), and some hydrochemical parameters in the outlet water were monitored. With the obtained data, it is expected to uncover the mechanisms behind the formation of pathogens in groundwater that has infiltrated from agricultural soils.

## 2. Materials and methods

### 2.1. General approach

Flow-through columns packed with agricultural soils collected from the karst area of Yunnan province, China, were used to mimic runoff and infiltration of agricultural drainage in karst areas, and to evaluate changes in the total bacteria and total coliform populations during sustained water supply. A focus was placed on counting bacteria in the column effluent to concentrate on the mobile bacteria with a higher likelihood of affecting a human recipient. Along with population enumerations, pH and permanganate index (COD_Mn_) concentration profiles were monitored along the length of the columns to know the water acid–base properties and organic matter contents that may also affect pathogens.

To understand the temporal and spatial variation of pathogenic microorganisms in the groundwater infiltrated from the selected soils, a column experiment stimulating the natural condition was carried out. The column was filled with collected soils with undisturbed natural porosity, and the pH value of the inlet water was adjusted to 7.0. Then the leaching experiment was carried out at 25°C for 42 days. To examine the temperature, pH, as well as porosity effects on the populations of pathogenic microorganisms in the groundwater infiltrated from agricultural soils, an orthogonal experiment with 3 factors and 3 levels was designed.

### 2.2. Flow-through columns

Flow-through glass columns (60-cm long, 25-cm inner diameter) were equipped with five sampling ports located at 10, 20, 30, 40, and 50 cm from the bottom of the column. The columns were filled with 40 cm high in a translational manner (every 10 cm correspondingly) to simulate the natural state of the agricultural soils in a karst area by controlling the density and porosity. The trapping air bubbles were precluded. The columns were wrapped in aluminum foil to minimize algal growth. The feed reservoirs were 2-L bottles wrapped in aluminum foil. Sterile deionized water amended with some inorganic and organic matter (Wang, 2019) was served as rainwater and added to the columns by a peristaltic pump to produce a constant water table (10 cm above the soil top surface).

### 2.3. Orthogonal experiment design

Here, an orthogonal experimental design method was applied to discuss the air temperature, pH values of inlet flow, and soil porosity effects on the populations of pathogenic microorganisms in the groundwater infiltrated from agricultural soils. Temperature, pH, and porosity were determined as three factors of the orthogonal experiment and each factor had three levels. The levels of each factor were determined by the actual conditions in the study area. It was assumed that any two factors did not interact with each other. The orthogonal array of the nine experiments is shown in Table 1, designed according to the orthogonal design table *L*_9_ (3^3^).

**Table 1:** The orthogonal table with three factors and three levels.

Experimental numbers	Factors		
	Temperature (°C)	pH	Porosity
1	10	6	0.50
2	20	8	0.50
3	30	7	0.50
4	10	7	0.55
5	20	6	0.55
6	30	8	0.55
7	10	8	0.60
8	20	7	0.60
9	30	6	0.60

### 2.4. Sampling and analyses

The water samples were collected at different depths (i.e., 10/20/30/40 cm) of each column at 0, 1, 3, 7, 10, 13, 23, 31, 36, 38, and 41 days after the soil was saturated by the inlet water in the temporal and spatial variation experiment. While in the orthogonal experiments, the effluent samples were collected at different depths at 7 and 9 days. The collected samples were stored in 100 ml sterile glass bottles at 4°C before analyses.

The pH values and COD_Mn_ concentrations in the effluent were measured by the electrode method and spectrophotometric method, respectively, (Federation and Association, 2005).

The TBC values were enumerated using the plate count method (China NHCOTPSRO, 2006). Water sample (1 ml) was inoculated onto beef extract peptone AGAR medium (Base Bio, Hangzhou, China) plate and incubated for 24 h at 37°C. Then, the colony-forming units (CFU) in the plate were enumerated and gained the TBC values (CFU/mL).

The TCC values were enumerated using the multi-tube fermentation (MTF) method (China NHCOTPSRO, 2006). A series of tubes with appropriate decimal dilutions of the water sample and lactose broth (Aobox, Beijing, China) were inoculated. Production of gas, acid formation, or abundant growth in the test tubes after 24 h of incubation at 37°C constitutes a positive presumptive reaction. All tubes with a positive presumptive reaction are subsequently subjected to a confirmation test. The formation of gas in a brilliant green lactose bile broth fermentation tube at any time within 24 h at 37°C constitutes a positive confirmation test. The results of the MTF technique are expressed in terms of the most probable number (MPN) of microorganisms present in 100 ml samples (MPN/100 ml).

### 2.5. Statistical evaluation

The statistical analysis was carried out in the statistical packages Origin Pro 8 and SPSS 19. Box plots were obtained from Origin Pro 8 to show each group’s data distribution, such as median and interquartile values. Analysis of variance (ANOVA) and Dunnett’s T3 pairwise comparison test in SPSS 19 were conducted to determine significant differences between individual treatments and to interpret the orthogonal experiments which were used to determine the significance of factors. A difference with *p* ≤ 0.05 was considered statistically significant. The Pearson’s correlation analysis in SPSS 19 was used to measure the relationships between every two groups.

## 3. Results

### 3.1. TBC and TCC in different soil depths and leaching times

**Figure 1:**
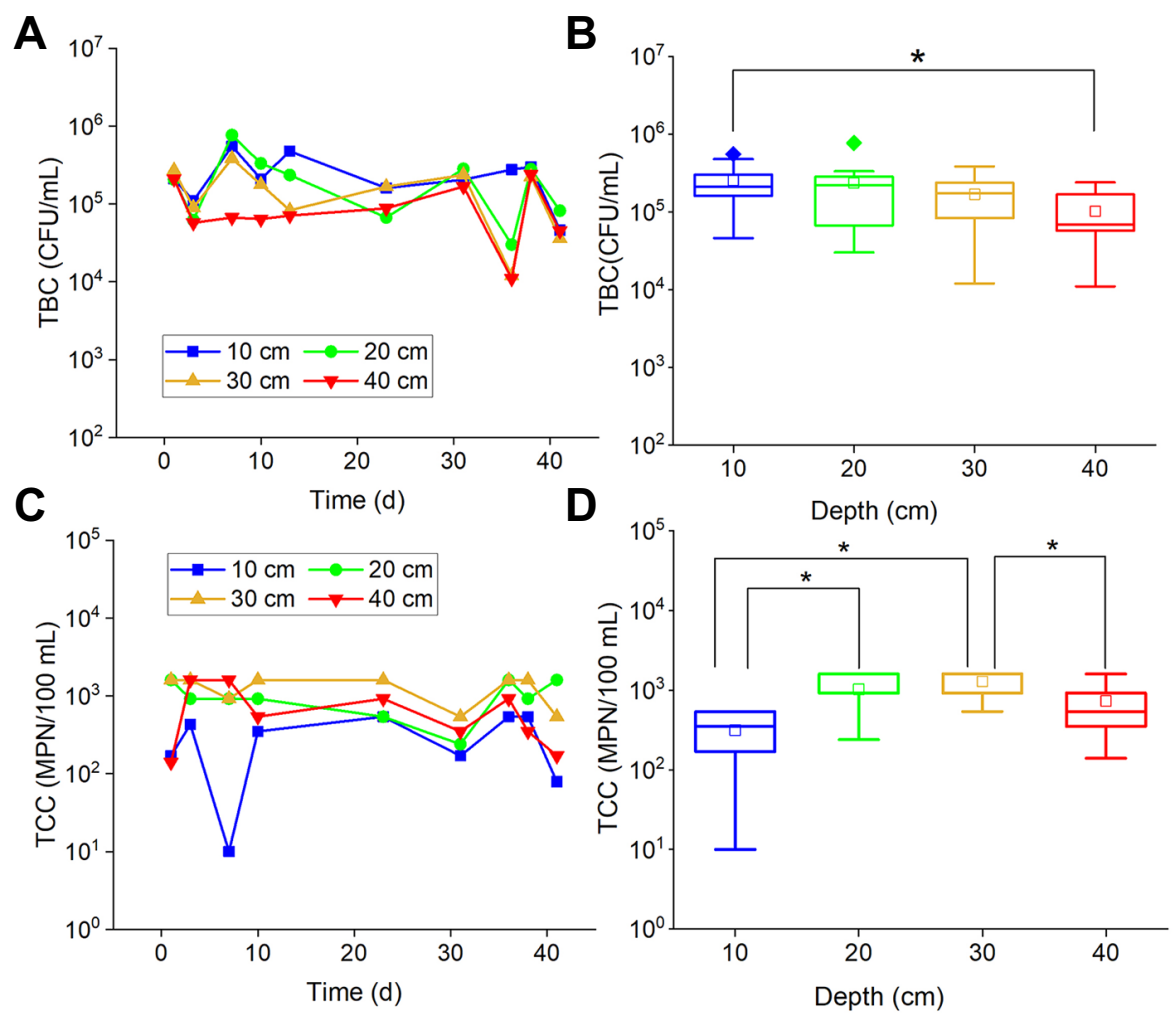
The variations of TBC and TCC with **(A,C)** time and **(B,D)** depth. The star (*) stands for there was a significant difference between the two concentrations (*p* < 0.05). Different colors stand for different depths. The central line and square of each box are the median and mean values, respectively, while the top and bottom of each box represent the third and first quartile, respectively. Whiskers on the box plot represent maximum and minimum values, while rhombuses above or below the box plots show outliers in the data.

Total bacteria count values in all examined depths fluctuated with time (Figure 1A), and most of them varied within the same order of magnitude (about 10^5^ CFU/ml). Similarly, the TCC points also fluctuated within the same order of magnitude (about 10^3^ MPN/100 ml) (Figure 1C). There was no obvious attenuation or increase of TBC and TCC in the period of more than 40 days of experiments. It is suggested that a majority of the bacteria and coliforms may persist and remain in a relatively stable state in the karst soils for an extended period of time. TBC values in different depths had no significant difference, except for TBC values at 10 and 40 cm (Figure 1B). The TBC values in 40 cm were slightly less than the 30 cm values. It has been suggested that the shallow soils in the karst region, with a thickness of less than 40 cm, may not effectively block the migration of bacteria into the groundwater. As for TCC, the values in 10 cm depth were significantly smaller than the values in 20 cm (*p* < 0.001) and 30 cm (*p* < 0.001), while the values in 40 cm depth were significantly (*p* < 0.001) smaller than the values in 30 cm (Figure 1D). This result suggested that the soils may have little impact on preventing coliforms from entering the groundwater.

### 3.2. The results of the orthogonal experiments

**Figure 2:**
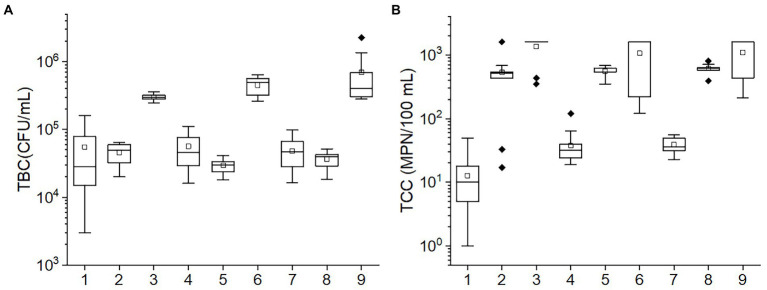
The box plots of the **(A)** TBC and **(B)** TCC concentrations in various conditions in the orthogonal experiment. The numbers in the horizontal axis stand for different conditions: 1: 10°C, pH = 6, porosity = 0.50; 2: 20°C, pH = 8, porosity = 0.50; 3: 30°C, pH = 7, porosity = 0.50; 4: 10°C, pH = 7, porosity = 0.55; 5: 20°C, pH = 6, porosity = 0.55; 6: 30°C, pH = 8, porosity = 0.55; 7: 10°C, pH = 8, porosity = 0.60; 8: 20°C, pH = 7, porosity = 0.60; 9: 30°C, pH = 6, porosity = 0.60. The central line and square of each box are the median and mean values, respectively, while the top and bottom of each box represent the third and first quartile, respectively. Whiskers on the box plot represent maximum and minimum values, while rhombuses above or below the box plots show outliers in the data.

The TBC and TCC values in various conditions according to the orthogonal experiment are shown in Figure 2. The TBC values in the 3, 6, and 9 tests and the TCC values in the 1, 4, and 7 tests were greater than others. Among temperature, pH, and porosity, temperature was found to have consistent values in tests 3, 6, and 9 and also in tests 1, 4, and 7. This suggests that temperature may be the primary factor impacting the bacteria community. The results of ANOVA also corroborated the speculation. The *p-*values of TBC in the test of between-subjects effects for temperature (<0.001) were the lowest relative to pH, and porosity (0.033 and 0.059, respectively). Likewise, for TCC, *p-*values of <0.001, 0.530, and 0.698 were observed for temperature, pH, and porosity, respectively, suggesting that both TBC and TCC were mainly affected by temperature, followed by pH and porosity.

### 3.3. Temperature effect

**Figure 3:**
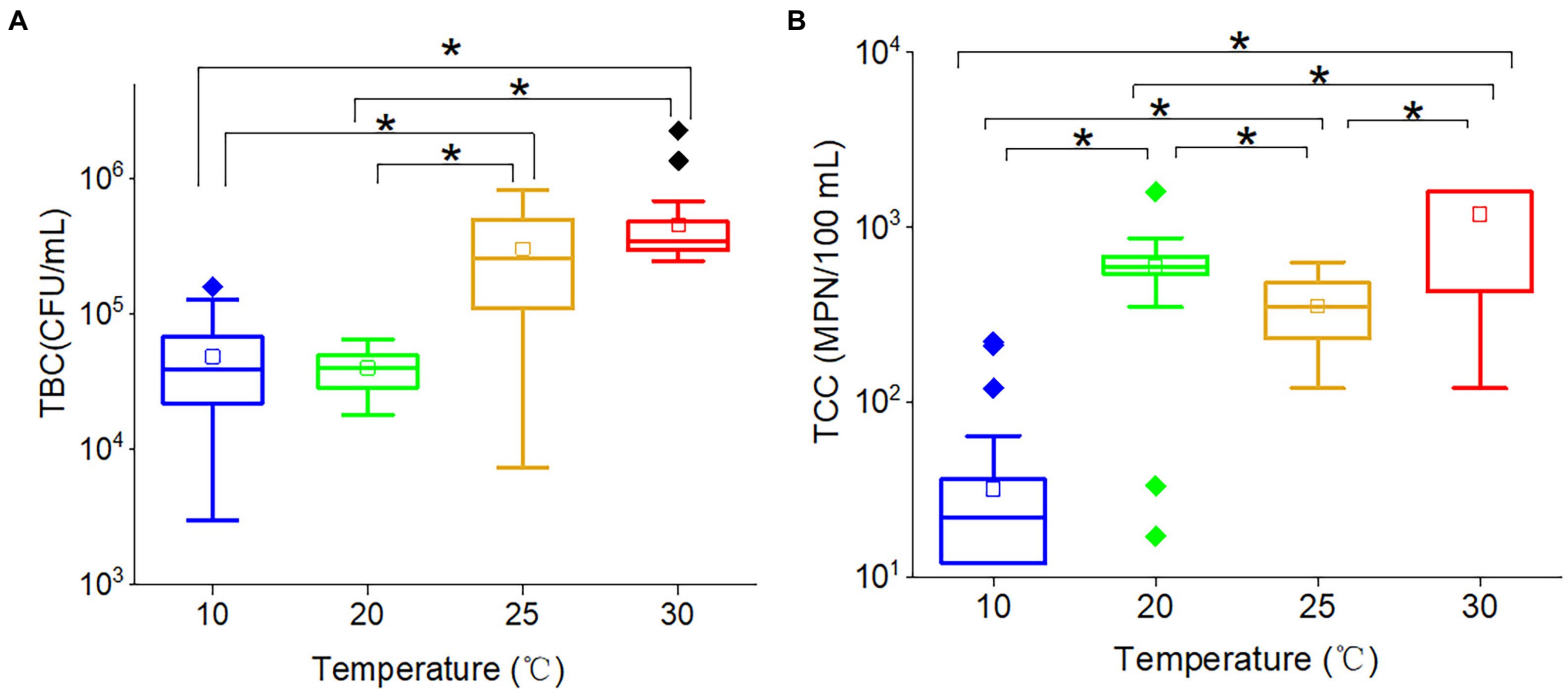
The box plots of **(A)** TBC and **(B)** TCC in various temperatures. The star (*) stands for there was a significant difference between the two concentrations (*p* < 0.05). Different colors stand for different temperatures. The central line and square of each box are the median and mean values, respectively, while the top and bottom of each box represent the third and first quartile, respectively. Whiskers on the box plot represent maximum and minimum values, while rhombuses above or below the box plots show outliers in the data.

Total bacteria count (*r* = 0.570, *p* < 0.001) and TCC (*r* = 0.677, *p* < 0.001) were significantly positively correlated with temperature, indicating that increased temperature would promote the proliferation of bacteria and coliforms. However, the effects of temperature on TBC and TCC were non-linear. For TBC (Figure 3A), there was no significant difference between 10 and 20°C, and the same trend was found between 25 and 30°C. The TBC at 25 and 30°C were significantly greater than at both 10 and 20°C. These results suggested that 20 ~ 25°C was potentially TBC-sensitive temperature, and temperature would not affect the TBC if the temperature varied between 10 and 20°C or between 25 and 30°C. The wide range of TBC concentrations observed at 25°C suggested that this temperature acted as a transitional point for bacteria, with some becoming active and others remaining inactive. While the TCC (Figure 3B) at 10, 20, 25, and 30°C were all significantly different from each other. The average TCC followed the order: 30°C (1,189 MPN/100 ml) > 20°C (354 MPN/100 ml) > 25°C (594 MPN/100 ml) > 10°C (32 MPN/100 ml). The TCC at 20°C and 25°C were in the same order of magnitude. Therefore, the results showed a positive correlation between temperature and TCC values, with higher temperatures resulting in higher TCC. The results suggested that the propagation of coliforms was also temperature dependent.

### 3.4. pH effect

**Figure 4:**
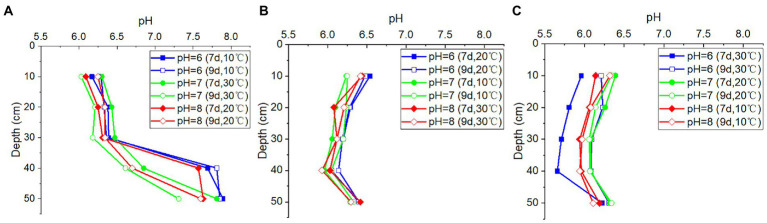
The pH values in outflow water from **(A)** column I (porosity = 0.5), **(B)** column II (porosity = 0.55), and **(C)** column III (porosity = 0.60) at different pH values of inlet water, depths, duration time, and temperatures.

Despite the noticeable differences in pH levels of the recharged water, the pH values in the outflow water from the soil column at the same depth remained nearly constant, regardless of temperature variations and the duration of time (Figure 4). This indicated that the variations in precipitation or other recharge would not significantly alter the groundwater pH, suggesting that the soil above the rocks had appreciable buffer capacity. However, the pH values were found to vary with the depths of the soil columns, indicating that the soil characteristics may impact the pH values of outflow water.

**Figure 5:**
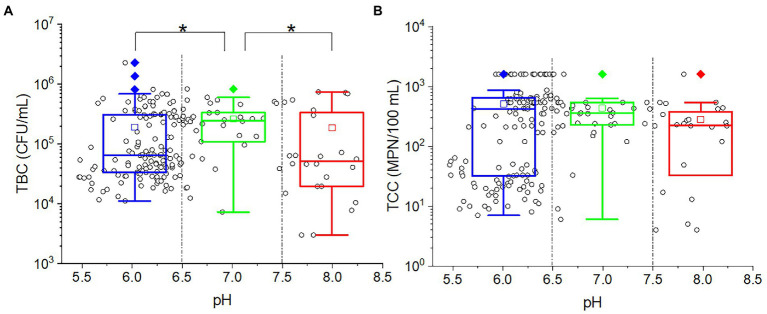
The relationship between **(A)** TBC [or **(B)** TCC] and pH. The cycles in the plots represent the measured data of each sample. The star (*) stands for there was a significant difference between the two concentrations (*p* < 0.05). Different colors stand for different pH values in the effluent. The central line and square of each box are the median and mean values, respectively, while the top and bottom of each box represent the third and first quartile, respectively. Whiskers on the box plot represent maximum and minimum values, while rhombuses above or below the box plots show outliers in the data.

As shown from the results, studying the impact of different pH levels on microorganisms can be challenging due to the varying pH recharge. In order to find out the pH effect on TBC and TCC, the outflow water samples were taken into consideration. There was no significate correlation between pH and TBC (*p* = 0.566) or TCC (*p* = 0.542). As demonstrated in Figure 5, plot the TBC and TCC against pH. Most of the points were located at a position of pH less than 6.5, indicating that most of the soils were weakly acidic. The outflow water samples were divided into three groups: low (pH ≤ 6.5), intermediate (6.5 < pH < 7.5) high (pH ≥ 7.5). In the intermediate pH group, more points were located at the high values of both TBC (greater than 10^5^ CFU/ml) and TCC (greater than 10^2^ MPN/100 ml). In the low-pH group, for both TBC and TCC, the points were scattered in both high values and low values. In the high-pH group, more points were concentrated in low values for TBC, while more points were concentrated in high values for TCC. Taking the points in each group together for statistical analysis, the TBC values in the middle pH group were significantly greater than in the low-pH group (*p* = 0.007) and high-pH group (*p* = 0.002), while for TCC, there was no significant difference among the three groups.

### 3.5. Porosity effect

**Figure 6:**
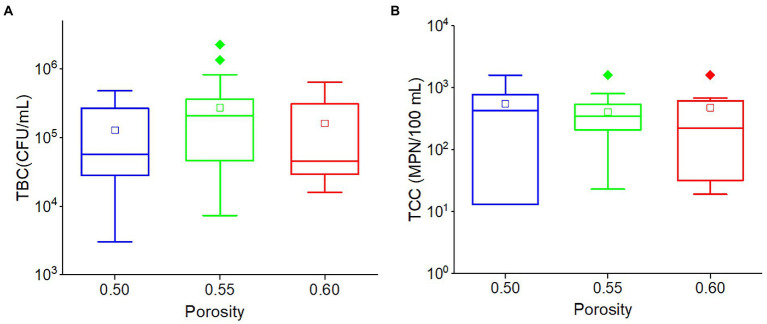
The box plots of **(A)** TBC and **(B)** TCC in various porosities. Different colors stand for different porosities. The central line and square of each box are the median and mean values, respectively, while the top and bottom of each box represent the third and first quartile, respectively. Whiskers on the box plot represent maximum and minimum values, while rhombuses above or below the box plots show outliers in the data.

There was no significant relationship between porosity and TBC (*r* = 0.102, *p* = 0.148) or TCC (*r* = −0.078, *p* = 0.274). Although the average TBC value in the outflow of 0.55 porosity soil was greater than others, and the average TCC value in the outflow of 0.50 porosity soil was greater than others, there was no significant difference in TBC and TCC values among the three porosities (0.50, 0.55, and 0.60) soils (Figure 6). This indicated that porosity would have a limited impact on TCC and TBC levels in the outflow water in this karst region.

### 3.6. The relationship between TBC and TCC

**Figure 7:**
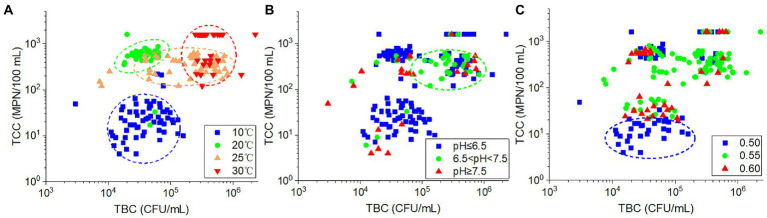
The relationship between TCC and TBC according to different **(A)** ambient temperatures, **(B)** effluent pH values, and **(C)** soil porosities.

The TCC values were significantly positively correlated with TBC (*r* = 0.365, *p* < 0.001). Among the plots of the relationship between TBC and TCC values, the samples collected at the same temperature were grouped together (Figure 7A). Both the TBC and TCC displayed lower values at 10°C and more at 30°C. At 20°C, only higher TCC was found, and this resulted in higher ratios of TCC to TBC. While at 25°C, the TBC values increased with nearly constant TCC values compared with those at 20°C. For the impact of pH, as shown in Figure 7B, the samples with low-pH values (pH ≤ 6.5) and high-pH values (pH ≥ 7.5) were dispersed, whereas the majority of samples with intermediate pH values (6.5 < pH < 7.5) were concentrated in an area of higher TCC and TBC. With regard to porosity (Figure 7C), samples with lower TCC value (in the cycle of the plot) were mostly found in soils with 0.5 porosity. The remaining samples were dispersed throughout the plot, indicating a lack of clear relationship between porosity and TBC or TCC in these samples. As a result, the variation of TBC and TCC in the samples appears to be influenced by temperature, pH, and porosity in a similar but also somewhat diverse manner.

## 4. Discussion

### 4.1. The temperature effect on microorganisms

As mentioned in the introduction, the temperature, pH, and porosity are considered to be the critical factors impacting the pathogen in the karst soil. The results of the present study have demonstrated that temperature was the most significant factor. Previous studies have identified two distinct impacts of temperature on soil bacteria, particularly coliforms. The first one is that higher temperatures may increase the soil’s hydraulic conductivity, and promote the desorption of bacteria from soil particles, and resulted in increased concentrations of bacteria in groundwater (Acea et al., 1988; Gharabaghi et al., 2015). The other one is that high temperatures create unfavorable conditions for the bacteria, leading to a rapid decline in their numbers, which is the common trend (Blaustein et al., 2013; Chandrasena et al., 2014).

Our results evinced that both the TBC and TCC increased due to the elevated temperatures, which supported the first possibility. However, previous studies have found that the effect of temperature on adsorption and hydraulic conductivity is not as pronounced (Acea et al., 1988). It should be noted that soil is not only an aggregation consisting predominantly of particulate matter, but also a growth medium (Aleklett et al., 2018).Therefore, the higher TBC and TCC values observed at relatively higher temperatures could be mainly attributed to an increase in bacterial populations in the soil. In the column soils, there was enough organic matter (represented by COD_Mn_, see Supplementary Figure S1), which can meet the demand for carbon and energy sources for bacteria (Wen et al., 2022). Most the coliforms are always deemed as the prevalent commensal inhabitants of the gastrointestinal tracts of humans and warm-blooded animals (Allocati et al., 2013); however, there was no such animal in the soil. Previous studies revealed that the multiplying of some coliforms in the soil may be caused by the soil protozoan, such as *Acanthamoeba polyphaga*, in which the coliforms and other pathogens survive and replicate (Barker et al., 1999). The elevated temperature stimulated the growth of bacteria (Kirchman et al., 2005), as well as protozoan (Müller and Geller, 1993), and may also indirectly stimulate the presence of coliforms.

It is generally acknowledged that 37°C is the optimum temperature for coliforms and some bacteria, and therefore, in most cases, the TBC and TCC increased with the elevating temperature in the laboratory medium (Jones et al., 1987; Raghubeer and Matches, 1990). However, it was not the case in the present study. Accordingly, the TBC and TCC increased with elevated temperature asynchronously. The TBC concentrations almost reached a maximum at 25°C, whereas the TCC peaked at 20°C. The phenomena may be caused by the protozoa in the soils. The optimum temperature for most protozoa is 18 ~ 25°C (Littleford, 1960). We speculated that at 20°C, the protozoa thrived, while the bacteria including coliforms were being preyed by the thriving protozoa (Bott and Kaplan, 1990). Most bacteria and some coliforms are digested as a food source, while most coliforms are able to resist lysosomal attack and therefore multiplied within membrane-bound vacuoles (Barker et al., 1999; Van Elsas et al., 2011; George et al., 2020; Mungroo et al., 2021). Previous studies have noted that protozoa, such as *Acanthamoeba,* can inhibit bacterial growth through the release of free radicals in their lysates (Connor et al., 1993), but have no inhibitory effect on certain coliforms, such as *E. coli*. On the contrary, those protozoa are an abundant source of amino acids, enzymes, fatty acids, and lipids and clearly provided coliforms with nutrients for growth (Van Elsas et al., 2011; Geisen et al., 2018; Gambushe et al., 2022). Therefore, the thriving of protozoa would lead to an increase in the number of coliforms and a decrease in other bacteria that are susceptible to predation by protozoa in the soil environment (Matin and Jung, 2011; Iqbal et al., 2014). This is consistent with our findings of lower TBC and higher TCC values at 20°C. Another possible reason is that some environmentally adapted coliforms may thrive at ambient temperatures (about 20°C) other than other temperatures.

### 4.2. The pH and porosity effects on microorganisms

Several studies illustrate that higher pH values produced longer survival time for coliforms in acidic soil, especially in south China (Jiang et al., 2002; Wang et al., 2014; Xing et al., 2019), while pH may negatively affect the persistence of the strains in soils with pH greater than 7 (Ma et al., 2012). Therefore, a neutral pH is desirable for most bacteria and protozoa (Rousk et al., 2010). In the present study, the intermediate pH groups (6.5 < pH < 7.5) had relatively high TBC and TCC, which exemplified the theory. Additionally, pH may affect the desorption of attached bacteria in the soil. Several studies found that bacterial retention in the columns was greatest in acidic soil and decreased with increasing pH (Scholl et al., 1990; Scholl and Harvey, 1992). However, the dissolved organic matter has been shown to alter the surface charge on suspended particulate matter, and therefore change the behavior of sorption (Scholl and Harvey, 1992). The wide range of dissolved organic matter (represented by COD_Mn_, see Supplementary Figure S1) in the column water might account for the wide range of values observed for both TBC and TCC. Combined with other factors, such as temperature, the pH effect was obscured in low-pH and high-pH groups’ samples.

The porosity variation altered the permeability of the soil. Generally, for the same texture soil, the more porosity, the greater the permeability coefficient. Under the same hydraulic gradient, the groundwater flow velocity in the greater porosity soil would be greater, and therefore, the water infiltrated through the greater porosity soil would have a higher concentration of bacteria (Smith et al., 1985; Huysman and Verstraete, 1993; Sepehrnia et al., 2019; Liu et al., 2020). In the present study, the lower TCC value samples were almost collected in the 0.5 porosity soils at 10°C, indicating that the soils with less porosity would retard the migration of coliforms, which could be observed at low temperature, while at high temperature, the retardation effect might be obscured by temperature effect. Therefore, there was no significant difference in TBC and TCC among different porosity soils.

Therefore, the pH and porosity might have affected the behavior of bacteria including coliforms in the karst soils, but the affected variations of TBC and TCC might be covered by the temperature effect.

### 4.3. The origination of the microorganisms in the groundwater

Previous studies on microorganism migration in aquifers have primarily centered that the microorganisms come from the soil surface and thus, when conducting related studies, these bacteria or similar organisms are added to the soil. Typically, the added microorganisms were retarded by the adsorption of soil and resulted in fewer microorganisms in outflow of groundwater (Castro and Tufenkji, 2007; Passmore et al., 2010; Wen et al., 2016; Bandy et al., 2018). However, in the present study, the outflow of groundwater infiltrated from the soil column contained higher level of TBC and TCC than the inlet water, indicating that the collected soil in the karst area may be a source of bacteria in the groundwater.

The TBC and TCC values in the outflow remained relatively unchanged for over 40 days, demonstrating that the bacteria, including the coliforms, remained active throughout that time in the soil. This is in contrast to prior studies, where coliform counts were observed to decrease significantly within a few days in various soils (Fremaux et al., 2008). Previous studies revealed that most bacteria concentrations would decline in several days if the soil condition was not suitable (Acea et al., 1988). However, in the present study, there was no introduction of foreign organisms and the environmental conditions remained relatively constant, providing a suitable environment for the survival of the studied bacteria.

Through the discussion about the temperature effect, only the survival of the bacteria cannot fully explain the phenomena of the study, the multiplied bacteria may be the main contributor of TBC and TCC in the outflow water. The TBC or TCC values in different depths were mostly in the same order of magnitude, indicating that the bacteria migration with groundwater flow played negligible roles for bacterial concentrations. Instead, the bacteria in the outflow water may stem from the thin-layer soils near the outlet.

Therefore, the soil in the study area may serve as both reservoirs and incubators for pathogenic bacteria. These bacteria can easily spread into the underground water, becoming a significant contributor to the contamination of groundwater pathogens.

### 4.4. Implication

The thin soil in the karst cannot effectively block coliforms and other pathogens from infiltrating groundwater through the seepage of rainfall. Instead, the soils served as both reservoirs and incubators for pathogenic bacteria. Accordingly, to reduce the pathogens concentrations in the infiltrated water, several approaches can be carried out, including slowing down the migration of pathogens, reducing the input of pathogens, and changing the environmental condition to inhibit the growth and multiplying of pathogens. In the soils, as discussed above, the migration velocity was not the primary factor for introducing bacteria to the groundwater, and therefore changing the structure of the soil, such as compressing or loosening the soil, would not reduce the TBC and TCC values. Whereas the multiplying of pathogens played a crucial role in controlling the TBC and TCC. Therefore, creating an uninviting and inhospitable environment for pathogens’ growth would be beneficial. For example, by acidizing or alkalizing the soil, or reducing the amount of organic matter input when planting crops.

Our results suggested that elevated temperature may help for breeding more pathogens. Therefore, in similar areas, it is vital to pay closer attention to the pathogens in areas with similar climate during hot weather, especially in the summer days.

## 5. Conclusion

In the study, the orthogonality column experiments were used to investigate how environmental factors, including ambient temperature, pH values of inlet water, and soil porosities, affect the microbiological indices in the leachate of agricultural soils from karst areas of Yunnan province, China. Results revealed that ambient temperature was the most crucial factor that affected the effluent bacteria, with higher temperature leading to the greater values of TBC and TCC. Although the neutral pH appeared to be suitable for the survival of bacteria, and the lower porosity may block the coliforms into the effluent, they did not significantly impact the bacteria. There was no obvious decay of TBC and TCC in the effluent for more than 40 days, indicating the bacteria including the coliforms remained active in the soils. As temperature increased, the TBC and TCC also increased, suggesting that agricultural soils in the study area likely served as both reservoirs and incubators for bacteria including coliforms, which may readily migrate into the groundwater and become an important source of groundwater pathogen contamination. The behavior of the bacteria in the soil with water seepage is intricate, and tightly related to the soil characteristics, hydrogeology condition, as well as other factors. In the present study, we suspected that temperature was the key factor for pathogens microorganisms, and protozoa might play a role in multiplying some pathogens. To validate the speculation, future studies, such as batch experiments, microscopic imaging, as well as metagenomic analysis, should be carried out.

## Data availability statement

The raw data supporting the conclusions of this article will be made available by the authors, without undue reservation.

## Author contributions

SW and MZ contributed to the conception and design of the study. SW carried out the experiments. ZN and CG performed the statistical analysis and wrote the first draft of the manuscript. All authors contributed to the manuscript revision, read, and approved the submitted version.

## Funding

This research was supported by Hebei Natural Science Foundation, D2022504009.

## Conflict of interest

The authors declare that the research was conducted in the absence of any commercial or financial relationships that could be construed as a potential conflict of interest.

The handling editor QL declared a shared affiliation with the authors at the time of review.

## Publisher’s note

All claims expressed in this article are solely those of the authors and do not necessarily represent those of their affiliated organizations, or those of the publisher, the editors and the reviewers. Any product that may be evaluated in this article, or claim that may be made by its manufacturer, is not guaranteed or endorsed by the publisher.
