# Supplementary figures and images for: The impact of environmental factors on the transport and survival of pathogens in agricultural soils from karst areas of Yunnan province, China: Laboratory column simulated leaching experiments
Source: Front Microbiol. 2023 Mar 16;14:1143900. doi: 10.3389/fmicb.2023.1143900 (PMC10060967; doi:10.3389/fmicb.2023.1143900)

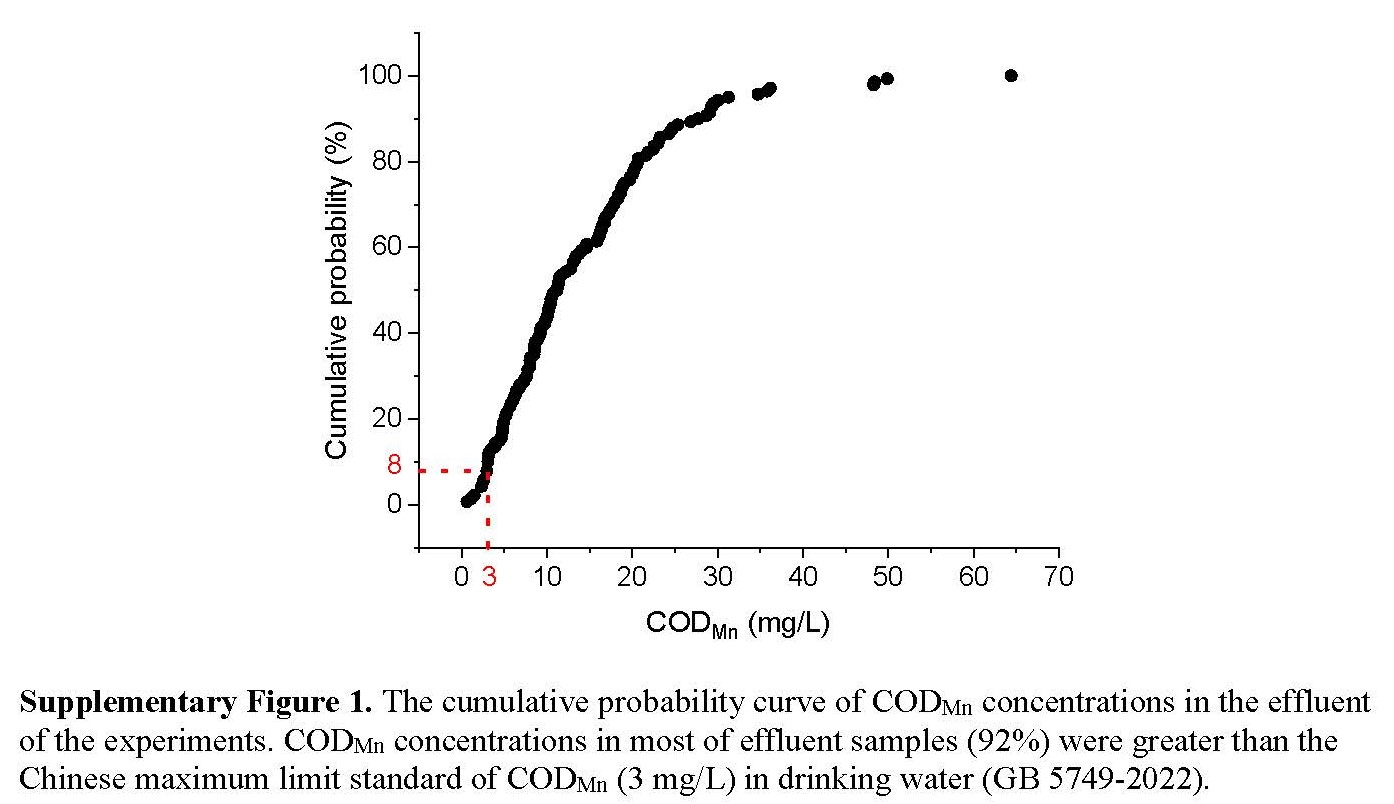

Supplement: Supplementary file 1 [file Image_1.JPEG]
